# Routine primary care screening for intimate partner violence and other adverse psychosocial exposures: what’s the evidence?

**DOI:** 10.1186/s12875-016-0500-5

**Published:** 2016-08-03

**Authors:** John D. McLennan, Harriet L. MacMillan

**Affiliations:** 1 Departments of Pediatrics, Psychiatry and Community Health Sciences, Cumming School of Medicine, University of Calgary, Calgary, Canada; 2 Departments of Psychiatry & Behavioural Neurosciences, and of Pediatrics, Faculty of Health Sciences, McMaster University, Hamilton, Canada

**Keywords:** Screening, Primary health care, Violence, Risk factors, Evidence-based medicine

## Abstract

**Background:**

Family physicians and other primary care practitioners are encouraged or expected to screen for an expanding array of concerns and problems including intimate partner violence (IPV). While there is no debate about the deleterious impact of violence and other adverse psychosocial exposures on health status, the key question raised here is about the value of routine screening in primary care for such exposures.

**Discussion:**

Several characteristics of IPV have led to consideration for routine IPV screening in primary care and during other healthcare encounters (e.g., emergency room visits) including: its high prevalence, concern that it may not be raised spontaneously if not prompted, and the burden of suffering associated with this exposure. Despite these factors, there are now three randomized controlled trials showing that screening does not reduce IPV or improve health outcomes. Yet, recommendations to routinely screen for IPV persist.

Similarly, adverse childhood experiences (ACEs) have several characteristics (e.g., high frequency, predictive power of such experiences for subsequent health problems, and concerns that they might not be identified without screening) suggesting they too should be considered for routine primary care screening. However, demonstration of strong associations with health outcomes, and even causality, do not necessarily translate into the benefits of routine screening for such experiences. To date, there have been no controlled trials examining the impact and outcomes – either beneficial or harmful - of routine ACEs screening. Even so, there is an expansion of calls for routine screening for ACEs.

**Summary:**

While we must prioritize how best to support and intervene with patients who have experienced IPV and other adverse psychosocial exposures, we should not be lulled into a false sense of security that our routine use of “screeners” results in better health outcomes or less violence without evidence for such. Decisions about implementation of routine screening for psychosocial concerns need similar rigorous debate and scrutiny of empirical evidence as that recommended for proposed physical health screening (e.g., for prostate and breast cancer).

## Background

Violence and other adverse psychosocial exposures are increasingly understood as common and important influences on health status. One key consideration is how such exposures can be easily identified within the context of primary care settings. To address this challenge, various screening tools have been developed and promoted for use in primary care. An essential question, however, is whether screening leads to actual health benefits.

There is no debate about the deleterious impact of violence and other adverse psychosocial exposures on health status, but the critical question remains: what is the value of routine screening in primary care for such exposures? The need to examine the evidence for this type of screening is just as critical as the ongoing research about the effectiveness of routine prostate specific antigen screening for prostate cancer or mammography screening for breast cancer. All the basic principles of screening apply, such as the accuracy of identification of a condition or exposure, the acceptability of the test or screen, evidence of health benefits for those screened positive, etc. [1].

Lessons could be learned from the careful considerations of the evidence and resulting recommendations regarding adult depression screening in primary care. Depression has a number of characteristics that support its consideration for primary care screening, including high prevalence, availability of easy-to-use screening tools, and effective interventions that could feasibly be delivered in primary care. Despite this, positive health outcomes have not been consistently demonstrated as a result of depression screening in primary care, but potential risks have been flagged (e.g., nocebo effects) [2]. These concerns contributed to the Canadian Task Force on Preventive Health Care (CTFPHC) recommending against routine depression screening in primary care in their most recent statement on screening for depression, a change from their 2005 recommendation [3].

The CTFPHC applied the Grading of Recommendations Assessment, Development and Evaluation (GRADE) approach in determining their recommendations [4]. In contrast, the US Preventive Service Task Force (USPSTF) uses a five level grading system with the top levels (A and B indicating high certainty that the net benefit is substantial or moderate, respectively) leading to USPSTF recommending a given strategy, with a C level leading to a selective or qualified recommendation [5]. The penultimate American Academy of Family Physicians’ (AAFP) recommendation for depression screening was informed by a 2009 USPSTF report [6] and included contextual considerations. More specifically, they issued a B grade such that screening is recommended “when staff-assisted depression care supports are in place,” but a Grade C, i.e., recommending against routine screening, when such supports are not in place [6]. In contrast, the 2016 recommendations from the USPSTF call for a more blanket B grade (i.e., without the above caveat), although the expanded text around this recommendation states that the “Screening should be implemented with adequate systems in place to ensure accurate diagnosis, effective treatment, and appropriate follow-up (p. 380) [7]. The AAFP has now adopted these new recommendations [8].

## Discussion

The World Health Organization (WHO) defines IPV as behaviour by a current or former intimate partner that causes physical, sexual or psychological harm, including acts of physical aggression, sexual coercion, psychological abuse, and controlling behaviours [9]. Several characteristics of IPV have led to consideration of routine IPV screening in primary care and during other healthcare encounters (e.g., emergency room visits) including: high prevalence, concern that issues may not be raised spontaneously if not prompted, and the burden of suffering associated with this exposure. Despite these factors, there are now three randomized controlled trials showing that routine IPV screening does not reduce IPV or improve health outcomes; one conducted in Canada with patients recruited from 26 health care settings followed for 18 months; a second carried out in a New Zealand emergency department with a three-month follow-up and the most recent, a US trial conducted in 10 primary health care centers with a 1 year follow-up [10–12].

Despite these RCT findings, the USPSTF has given a grade B rating and recommends that clinicians screen women of childbearing age for IPV [13]. In addition, promotion of this approach persists, including a mandate for such screening within the US Affordable Care Act [14]. It is not clear why the USPSTF does not appear to have applied the same considerations with regard to strength of evidence for IPV screening, as for other exposures or conditions [15].

The extent to which countries outside the US are engaged in, or promoting, routine IPV screening is not known. However, related recommendations are being made in at least some countries. For example, the UK National Institute for Health and Care Excellence, within an extensive list of recommendations on approaching domestic violence and abuse, recommends that “…trained staff in [a variety of healthcare areas] ask service users whether they have experienced domestic violence and abuse. This should be a routine part of good clinical practice, even when there are no indicators of such violence and abuse” (Recommendation 6) [16].

Other types of routine psychosocial risk factor screening are also being promoted. For example, there have been recent calls to screen for adverse childhood experiences (ACEs) in adults. This in part stems from a cross-sectional study of a US clinical sample demonstrating a graded association between increased number of ACEs and various adult health problems [17]. Proponents of such screening point to the high frequency of ACEs, the predictive power of such experiences for subsequent health problems, and concerns that they might not be identified without screening. However, demonstration of strong associations with health outcomes, and even causality, does not necessarily translate into benefits of routine screening for such factors and identification of those at risk for adverse health outcomes. To date there have been no controlled trials examining the impact and outcomes – either beneficial or harmful - of routine ACE screening.

The USPSTF has not provided a specific recommendation on ACE screening in primary care, however, in related work (which was updated in 2013), they recommend, for children who do not have signs or symptoms of maltreatment, that “the current evidence is insufficient to assess the balance of benefits and harms of primary care interventions to prevent child maltreatment” [18]. Nevertheless, calls for implementing ACE screening persist (e.g., a bill [H.299] before the Vermont General Assembly proposing widespread use of ACE screening in that state) [19]. Of note, although the World Health Organization (WHO) is currently promoting the evaluation of an international version of the ACE instrument, it does not recommend ACE screening as a part of health services at this time [20].

Even in situations where the lack of evidence for health benefits of screening for either IPV or child maltreatment is acknowledged, a rationale for its use may be framed as “it’s better than doing nothing”, “it doesn’t hurt”, or “it’s important to increase awareness”. Rationales such as these are problematic for several reasons. There are finite resources to address the many competing demands in primary care. Choosing to implement a given screening tool (and then dealing with the response) entails “opportunity costs,” i.e., “by choosing to use available resources in one way, we forgo other opportunities to use these same resources” (p. 153) [21]. These activities may preclude addressing other health issues or concerns raised by a patient. Additional costs, beyond screening, may occur as a function of responses to false positives. Unfortunately, there appears to be little evaluation of trade-offs and considerations of relative cost-benefits when it comes to these and other psychosocial screening recommendations.

Beyond opportunity costs, risks for direct harm need to be considered, including poor responses to true positives and inappropriate responses to false positives. For example, if screening correctly identifies IPV in a woman with children and this leads to involvement of child protection services with an investigation that results in the perpetrator becoming aware of the process, this may place the woman at greater risk of IPV exposure. In the case of a false positive, a related harm could be a costly and stressful child protection investigation where none was warranted. If such risks are not addressed and there are no net benefits to the screening, net harm is possible. As some screeners may lead to over-identification [22], such concerns may be more than theoretical.

Even among those correctly identified through screening, an important ethical issue is raised if such identification is not “backed-up” with clear clinical pathways from identification to effective interventions. In the American Academy of Pediatrics’ call for an expanded role for pediatricians in addressing ACEs, there is an acknowledgement that there is “relatively limited availability of evidence-based strategies …shown to reduce sources of toxic stress in the lives of young children or mitigate their adverse consequences” (p.e226) and that “routine screening for increased vulnerability is useful only if collaborative relationships exist with local services to address the identified concerns…it is also essential that those services demonstrate evidence of effectiveness” (p.e227) [23]. They appropriately call for prioritizing investment in evaluation of promising interventions [23], hopefully as a pre-requisite to routine screening.

## Summary

While we must prioritize how best to support and intervene with patients who have experienced IPV and other adverse psychosocial exposures, we should not be lulled into a false sense of security that our routine use of “screeners” results in better health outcomes or less violence without evidence. Better mechanisms and regulations are needed to ensure critical evaluations are performed, and minimal standards are achieved, for proposed screening before dissemination. In the case of IPV, international guidelines from the WHO have now moved beyond the concept of screening to instead focus on safe, caring and effective case finding linked to available services [24].

## Conclusion

As is evident from the IPV literature, it has been essential to examine the impact of screening, rather than focusing only on how to screen, based on the erroneous assumption that accurate screening will inevitably lead to positive impacts. It is clear that decisions about any and all health care screening, including within the context of primary care, should be based on evidence. The Choosing Wisely® initiative is one approach that makes explicit recommendations on screening as well as other medical procedures [25]. The AAFP is one of the many partners in this initiative. Within their Choosing Wisely recommendations, the AAFP provides an explicit statement around prostate cancer screening, but there are no directives around IPV or other adverse psychosocial exposure screening. Hopefully such topics will be addressed as part of the Choosing Wisely recommendations in the near future. In the meantime, family physicians and other primary care practitioners should be alert to the clinical features associated with IPV; inquiry about IPV exposure should occur in the context of case finding and diagnostic assessment by clinicians competent to do so and able to respond appropriately through referral and/or follow up. A similar approach is advocated for other types of violence exposure such as child maltreatment. It is essential that such inquiry take into account issues of safety and avoidance of harm. Decisions about implementation of approaches to identify experiences of violence such as IPV, warrant the same level of rigorous evaluation as other exposures and health problems.
